# Large-Scale Internet of Things Multi-Sensor Measurement Node for Smart Grid Enhancement

**DOI:** 10.3390/s21238093

**Published:** 2021-12-03

**Authors:** Adrian I. Petrariu, Eugen Coca, Alexandru Lavric

**Affiliations:** 1 Computers, Electronics and Automation Department, Stefan cel Mare University of Suceava, 720229 Suceava, Romania; eugen.coca@usv.ro (E.C.); lavric@usm.ro (A.L.); 2 MANSiD Research Center, Stefan cel Mare University of Suceava, 720229 Suceava, Romania

**Keywords:** smart grid, LoRaWAN, partial discharge, predictive maintenance, scalability

## Abstract

Electric power infrastructure has revolutionized our world and our way of living has completely changed. The necessary amount of energy is increasing faster than we realize. In these conditions, the grid is forced to run against its limitations, resulting in more frequent blackouts. Thus, urgent solutions need to be found to meet this greater and greater energy demand. By using the internet of things infrastructure, we can remotely manage distribution points, receiving data that can predict any future failure points on the grid. In this work, we present the design of a fully reconfigurable wireless sensor node that can sense the smart grid environment. The proposed prototype uses a modular developed hardware platform that can be easily integrated into the smart grid concept in a scalable manner and collects data using the LoRaWAN communication protocol. The designed architecture was tested for a period of 6 months, revealing the feasibility and scalability of the system, and opening new directions in the remote failure prediction of low voltage/medium voltage switchgears on the electric grid.

## 1. Introduction

Electric power infrastructure has revolutionized our world. With the advent of electricity, our way of living completely changed. But, as our energy needs increase faster than we realize, our energy sources depleted at a similar rate. Our grid is running against its limitations and blackout conditions are more frequently met. Due to the increase of greenhouse gas emission, our carbon footprint is also increasing, which is leading to climate change and numerous associated problems [1]. Therefore, a solution is needed to tackle all these problems. A solution is required for using electricity in a sustainable manner. We can use information technology to overhaul the electric grid and to monitor it [2]. With the ever-increasing demand of electrical vehicles, the real time monitoring and measurement of the power grid is mandatory [3]. Thus, urgent solutions need to be found as to meet this higher and higher energy demand.

The conventional grid consists of electromechanical components which cannot be controlled in real time. However, the smart grid comes into the picture when ICT (information and communication technology), electrical, and power systems are used collectively. In the existing grid, communication is one way; information goes from power generating units and utilities to the consumer, but almost never from the consumer to the utilities. The smart grid concept allows two-way communications with all stakeholders, so that information can be shared in a productive way. Utility companies may understand the consumption patterns of consumers and decide the price per electricity unit depending on peak loads and usage time.

The conventional grid does not integrate many sensors in its network, due to which it is considered blind, being unable to self-monitor and self-heal. That is why, much of the time, utility companies are not aware of failures or blackouts and cannot predict them so as to prepare for consequence to the end users. Testing and restoration tasks are also usually not automatic, involving human presence in multiple remote locations. On the other hand, in a smart grid framework, a few sensors are attached throughout the network, which enable it to self-monitor. By extending the concept, utility companies will be able to pick-up information from the network remotely and the network will have the capability to even self-heal in an automatic manner [4,5].

Analyzing the demands from the abovementioned, we can say that the main contribution of this work is the design of a fully reconfigurable wireless sensor node that can sense the smart grid environment. The proposed prototype can be easily integrated into the smart grid concept in a scalable manner, using a modular hardware platform developed by the authors.

In the specialized literature, there are a series of scientific papers which describe various smart grid architectures. This paper comes to fill in the gap by implementing and designing a smart grid architecture based a multi-sensor wireless node that uses state of the art technologies for sensing the environment. The designed concept allows the integration of a high-density of sensors distributed over a large-scale geographical area. This is possible by using LPWAN (Low-Power Wide-Area Network) technologies. Some of the most used technologies are SigFox, LoRaWAN, or NB-IoT. Of these, LoRaWAN is the most suitable for our scenario, due to its numerous advantages compared with the others, such as unlicensed spectrum transmitting, easy deployment by any hardware/software developer for both components, nodes, and gateways. SigFox is a technology that is only available in selected countries, being a mobile carrier supported solution, and requires subscription fees to register new nodes in the network. Furthermore, several message transmission restrictions are in place. Thus, only 140 messages of 12 bytes are possible for the uplink, and only four messages of eight bytes are possible for the downlink. These aspects limit the applicability of the SigFox communication protocol in different geographical regions. Meanwhile, NB-IoT operates in the license spectrum and is mostly driven by leading telecommunication companies around the world, so flexibility for the end-user is limited. Furthermore, to transmit messages, each node must be registered on the network, generating additional costs for each installed node.

The paper is organized as follows: first, a brief introduction related to the state-of-the-art, followed by Section 2, where the main challenges of the smart grid concept are presented. The LoRa modulation and the LoRaWAN communication protocol are discussed in Section 3. In Section 4, the smart grid architecture design is presented and analyzed with emphasis on the developed multi-sensor measurement node. The experimental results and discussion of them are presented in Section 5. The final section of the paper represents the conclusions and the overall performance evaluation of the proposed multi-sensor monitoring node.

## 2. Smart Grid Challenges and Solutions

The concepts of cyber-physical systems (CPS) [6] or the internet of things (IoT), which have been around for more than a decade now, are currently creating a great deal of buzz in the marketplace and media, with promises to enhance the way we live, travel and work. There are three major areas of IoT applications: in the consumer, industrial, and public sectors. Recent interest has mainly focused on the consumer side, including consumer appliances, home area networks, and other small office applications. Industrial applications promise to improve business outcomes for many sectors, including manufacturing, asset management, and healthcare. In the case of public sector applications, the internet of things is a major enabling concept to accelerate the development and deployment of smart city solutions, including electric vehicles charging stations [7,8,9], utilities monitoring, or public transport fleet management [10].

In the smart city concept, the most important element is represented by the electric grid, which basically transports electricity to each consumer. The electric grid parameters can be monitored using wired or wireless communication systems.

For wired communication, PLCs (power line communications) are the best option due to the working principle and use of the existing grid infrastructure. They are efficient if they are used in a small architecture configuration. In cases where two communication points are located between a transformer, PLC systems are useless due to the galvanic isolation between primary and secondary windings. Thus, to optimize communication, some hybrid solutions exist, where low-range wireless communications are attached to each transformer side PLC system to ensure data transmission. Other solutions are based on PLC and cellular technologies, extending the communication range between isolated power nodes, or using it to increase coverage of the non-signal or poor-signal areas encountered in on-site implementation [11,12,13]. These solutions have a monthly fee and are dependent on cellular coverage.

Below, some wireless communication solutions are briefly listed from the scientific literature, with applications for smart city and smart grid concepts.

In [14], the authors propose a method to detect the partial discharge that may occur into a switchgear by using a synchronization mechanism between multiple switchgears in a row with LoRa (long-range) technology and TEV (transient earth voltage) sensors. This method uses the attenuation principle in the process of electromagnetic wave propagation, so by comparing measured amplitude values received at each switchgear from the same discharge source, it can detect the faulty switchgear from a row. Thus, LoRa technology is used only to detect which switchgear is faulty or not, the information being transmitted and processed locally.

Gao et al. [15] use LPWAN technologies to transmit any grid issue that may occur in the medium voltage (MV) distribution power network. In the paper presented, an IoT-based HFCT (high frequency current transformer) sensor with LoRa and NB-IoT capabilities transmits any partial discharge issue from the MV cables.

Another hybrid approach is made in [16], where LoRa and SigFox technologies are used to communicate in a smart grid infrastructure. The authors propose a general architecture for a local implemented project called MAIGE. Several sensors are attached to some key points (high voltage/medium voltage switchgears or transformation centers) that transmit information regarding electrical discharge, grounding problems, or battery electrolyte levels to a SCADA (supervisory control and data acquisition) monitoring center.

The hybrid communication approach is tackled in other papers [17,18,19,20,21], combining the well-known and used LPWAN, ZigBee, or 5G communication systems to transmit smart grid issues to a control and command center.

**Figure 1:**
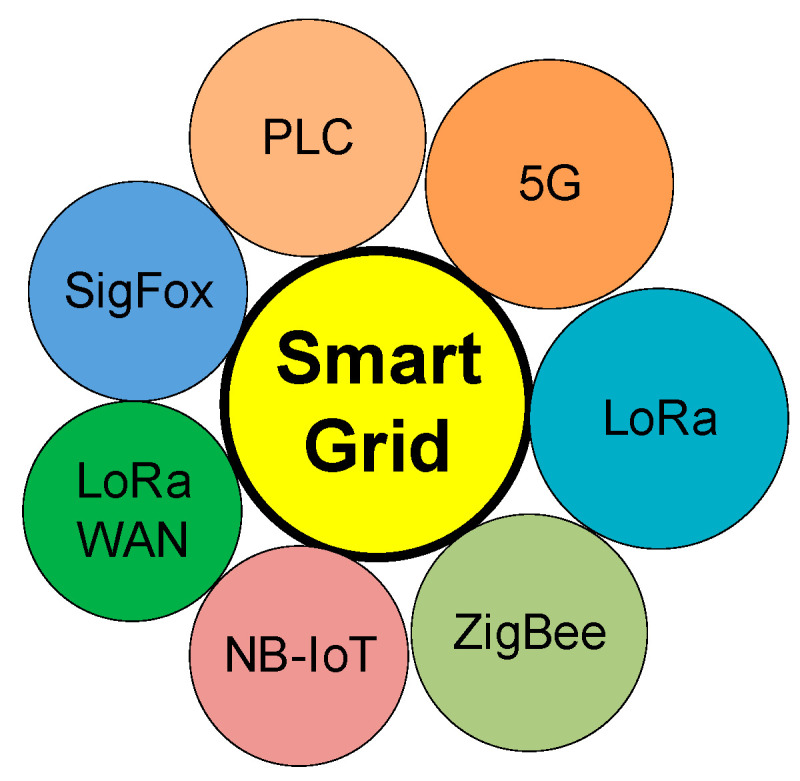
Technologies identified in the smart grid concept.

Figure 1 centralizes the main communication protocols that can be integrated in the smart grid: PLC, 5G, LoRa, ZigBee, LPWAN, NB-IoT, and LoRaWAN (Long-Range Wide Area Network) in a cost effective and scalable manner.

In this paper we present the design implementations and testing of a standalone smart WSN (wireless sensor node), called the multi-sensor measuring node (MSMN), that can be used to monitor different parameters from an electric grid, where remote monitoring of failures or blackouts in low voltage/medium voltage (LV/MV) switchgears is needed for grid efficiency management, This monitoring multisensory node can be integrated into a smart grid architecture using LoRa technology and can oversee power lines, detecting the eventual failure of the LV/MV switchgears in crowded cities. The proposed solution is called “multi-sensor” due to the use of different sensors for data acquisition, such as temperature, humidity, air pressure or ozone concentration, that are integrated into a modular hardware platform developed by the authors. Those sensors cannot be integrated into a single sensor type, so for our goal, we use more than one sensor, connected through different communication busses in the hardware platform. The obtained node has the main advantage of being flexible and easy to use, allowing the end user to add new sensors depending on the monitored environment. This reconfigurability and modularity of the proposed MSMN node entails cost efficiency and must be considered when evaluating its performance level. The high level of performance of the developed node is possible only by using the integrated acquisition algorithm, presented in detail in Section 4, where each individual sensor is controlled separately.

## 3. LoRa Technology Overview

LoRa is a wireless transmitting technology that used chirp spread spectrum (CSS) modulation, ensuring long-range communication links between modules. This type of modulation offers robustness against interferences and a very low signal-to-noise ratio (SNR) for the receiver to be able to demodulate the received signal. For wirelessly transmitting the information, LoRa uses the unlicensed radio spectrum with the ISM (Industrial, Scientific, Medical) or SRD (Short-Range Devices) band, using 433 MHz, 868 MHz, or 915 MHz as the main frequencies.

LoRa is a long-range technology [22] that has a communication range up to 10 km and 50 km in urban or rural areas, respectively, depending upon the spreading factor (SF) used. It is a low-power consumer (can use a few mA to hundreds of mA when transmitting) [23,24], is scalable (can be reconfigured remotely, depending on the infrastructure) [25,26], and does not generate costs during its lifetime. LoRa is thus a suitable solution for applications that require a very long battery lifetime and reduced cost.

Due to its unique modulation technique that implies CSS, LoRa technology allows users to negotiate between data rate and the maximum range by varying the spreading factor of the transmitter. The standard bandwidth is 125 kHz for the European region and 250 kHz for the US region.

Compared to other existing LPWAN technologies, LoRa uses the same phase between two chirp symbols. Thus, the synchronization mechanism between the node and the gateway is improved, resulting in a cheaper hardware for the gateway. Some related LoRa specifications are in Table 1, considering the 125 kHz bandwidth.

LoRa is the modulation technique used in the LoRaWAN systems. LoRaWAN uses long-range star architecture in which gateways are used to relay messages between the end nodes and a central core network, being a protocol for medium access control (MAC) designed for internet of things (IoT) applications. In a LoRaWAN architecture, the transmitters (nodes) are not associated with a specific gateway, their transmissions being received by all the gateways available in the communication range. Furthermore, LoRaWAN uses some specific communication algorithms that imply adaptive data rate (ADR) in coding rate (CR) and spreading factor estimations. Although it uses a free radio frequency band, some limitations are required in transmitting duty cycle, being imposed by the current international laws governed by the ETSI (European Telecommunications Standards Institute-Sophia-Antipolis, France) and FCC (Federal Communications Commission—Washington, DC, USA). Thus, a duty cycle less or equal than 1% is allowed for all European sub-channels.

## 4. Smart Grid Monitoring System

### 4.1. Monitoring Architecture

**Figure 2:**
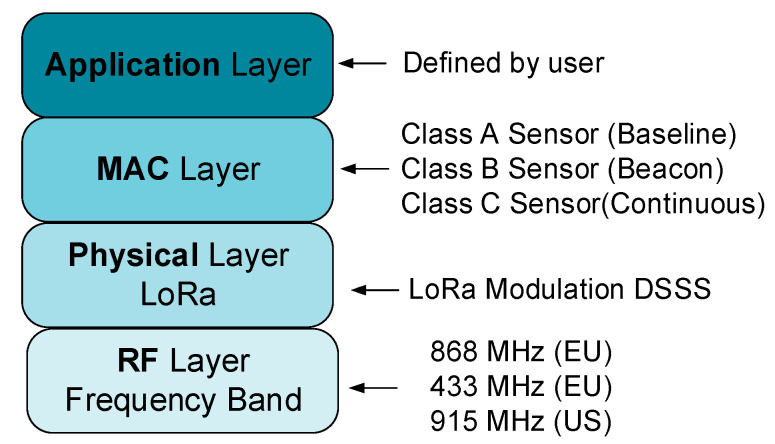
The LoRaWAN communication stack.

The proposed monitoring architecture from this paper uses the LoRaWAN specification. LoRaWAN is the communication protocol defined by the LoRa Alliance. Figure 2 presents the LoRaWAN communication stack. The physical layer of the protocol uses the LoRa modulation, discussed in the previous section, combined with a DSSS (direct sequence spread spectrum) techniques inspired from radar communication systems. The frequency band used belongs to ISM or SRD—thus, no license fees are needed—and it entails a cost reduction, which is a major advantage that LoRaWAN technology offers. The MAC (medium access control) layer of the LoRaWAN protocol defines three classes of sensors named class A, B, and C that determine different communication constraints regarding transmission time and transmission mode. Most of the sensor architectures for the smart grid concept use class C of the LoRaWAN sensor due to the existing electricity in the monitored center, which means continuous reception and a bidirectional link between the monitored point and the LoRaWAN gateway. In our proposed architecture, the multi-sensor node will be powered from a battery with a renewable energy source backup and the selected communication class will be A, to obtain energy efficiency for the whole system.

The LoRaWAN gateway acts as a relay between the smart grid and the internet network on which the information is transmitted. The information is stored and processed on a web server with the ability to display different reports and statistics. The main advantage of the proposed monitoring architecture is the decentralized data collection process with the ability of integrating an exceptionally large number of monitoring points.

**Figure 3:**
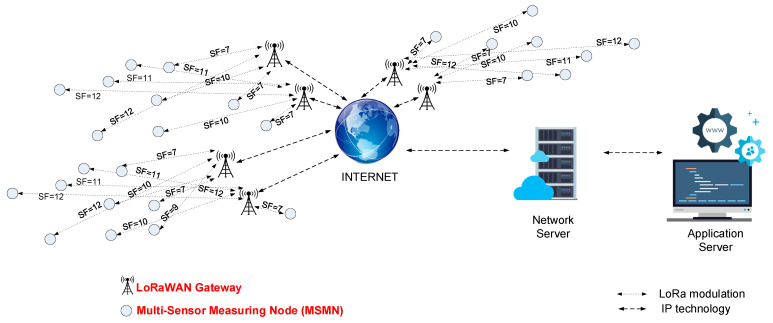
The proposed smart grid architecture based on the LoRaWAN communication.

Figure 3 presents the proposed smart grid architecture. The architecture is distributed over a large geographical area specific to urban non-LoS (line-of-sight) communication conditions. We can observe that the monitoring nodes that are located at the edge of the network are communicating using an SF set to 12. To increase the number of integrated measuring nodes, the star network topology is used, in which the sink node is represented by the LoRaWAN gateway.

The optimal placement of the sink node will be addressed in the next section of the paper using some measurements and with some simulation scenarios based on the RadioPlanner simulator.

Each multi sensor measuring node (MSMN) can sense the monitoring environment.

Advantages of the MSMN node:

− Modular platform with multi sensors integration capabilities: any type of analogue, digital, or I2C interfaced sensor;
− High reconfigurability of the developed platform, which means that new sensors can be easily added to the developed platform;
− The MSMN can be placed without any previous configuration in an area with LoRaWAN coverage and will automatically connect to the network;
− The MSMN node can function as standalone wireless module for a long period of time without any maintenance due to renewable energy integration;
− The MSMN node can be customized with other communication protocols based on the usability scenario.

When the MSMN node sends measurement data, the information is transmitted to the closest LoRaWAN gateway and can be processed by a monitoring center.

### 4.2. Multi-Sensor Monitoring Node

**Figure 4:**
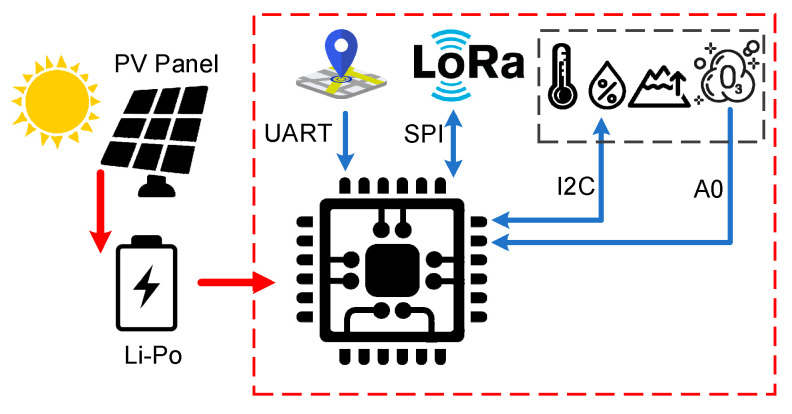
Proposed MSMN architecture.

The monitoring architecture uses the LoRaWAN specification. The general architecture of the proposed LoRaWAN node is listed in Figure 4.

The MSMN uses the platform proposed in [27] as the main board. This platform has a LoRa communication module to provide a long-distance communication and to cover the areas where other communication types like GSM/GPRS have a poor coverage. The MSMS also has a NEO-6G GPS module for location estimation. This module has a particular feature: a very low-power consumption of only 72 mW at a 1.8 V voltage rating. microcontroller, a ATmega88A is used, and the clock is set to 1 MHz; it works with a power supply from 1.8 V to a maximum of 3 V. All the external features of the platform are programmed to work in sleep mode. Thus, all the system architecture is low-power consumption, only demanding approximately 63 mA in active mode when SF7 is used or 172 mA when SF12 is used. In sleep mode, the entire platform drains only 10 µA.

The solution proposed in [27] is a modular one. Thus, miscellaneous sensors for data acquisition can be integrated on the main board. Environmental data acquisition sensors like air temperature, air humidity, air pressure, air quality (BME680 connected using I2C bus), and ozone (MQ-131 connected using A0 analogic input) are available in the architecture from the MSMN node. These sensors are required for the LV/MV (low voltage/medium voltage) switchgear monitoring, to avoid problems caused by malfunction of these electric energy distribution cells. According to some scientific papers [28,29,30], the main cause of electrical grid malfunction is the interruption of the power supply, due to damage that may occur in the LV/MV switchgear cells (which supply household users). These problems occur due to improper administration of the MV switchgear cells because in most of the cases they are not supervised, being mounted in the open field with different atmospheric conditions. Variable temperature and humidity can cause changes in the insulation of the conductors over time, which can lead to the corrosion of metal elements or even the appearance of the corona effect. If certain compartments in a switchgear are monitored, faults can be predicted, thus avoiding the problems mentioned above. Corona effect, which can be found as corona discharges, can be easily monitored because it is an electrical discharge caused by the ionization of a fluid such as air surrounding a conductor carrying a high voltage. In many high voltage applications, like MV switchgears where high voltages pass through electric wires, corona is an unwanted side effect. In the air, coronas generate gases such as ozone (O_3_) and nitric oxide (NO), and in turn, nitrogen dioxide (NO_2_) and thus nitric acid (HNO_3_) if water vapor is present. These gases are corrosive and can degrade and embrittle nearby materials such as the switchgear high voltage conductor’s isolation or even the metal case.

Water vapor can occur due to sudden changes in temperature and high humidity conditions. If a sensor is used to monitor the mentioned parameters, the value of the dew point can be determined exactly and thus the appearance of water vapor in a switchgear, which can cause corona discharge for instance, can be anticipated. In this case, the BME680 sensor is used, which through an I2C connection can transmit information related to temperature, humidity, air quality, and atmospheric pressure.

Thus, the sensors mentioned in the MSMN node can transmit data from the field, and decisions can be made at a monitoring center, history files can be created, or alarms can be set for the prevention of problems and the optimal management of LV/MV cells.

The power supply of the node is a rechargeable Li-Po battery with a capacity of 1000 mAh. This battery capacity is necessary mainly due to the ozone measuring sensor, MQ-131. According to the datasheet, the sensor is a metal oxide semiconductor (MOS) type gas sensor also known as a chemiresistor, because detection is based on the change of resistance of the sensing material when the gas encounters the material. Using a simple voltage divider network, the gas concentration can be easily detected. Thus, for good operation, the sensor heater resistance needs approximately 900 mW, consuming the most battery capacity.

To optimize the energy consumption of the node, the algorithm proposed in [27] is used. Firstly, the MCU, the BME680, and the MQ-131 sensors are woken up. To obtain the correct values, the MQ-131 heater resistance needs to preheat at least 2 min before obtaining the measurements. During this time, the BME680 sensor values are read and stored in the MCU internal EEPROM memory. The next step is to put the BM680 sensor in sleep mode and verify the NEO-6G GPS coordinates. For this to happen, an internal counter is checked to verify if a 24-h interval has elapsed from the last GPS fix measurement. If this condition is fulfilled, the GPS module is powered-up, otherwise this step is passed over. This process may take up to 26 s (according to the GPS datasheet) only once a day. After the GPS location coordinates are collected and saved, the GPS module is deactivated. The next step is acquiring the ozone values from the MQ-131 sensor and storing them in the same EEPROM memory. After that, all the integrated sensors are deactivated and at the same time the LoRa communication module is woken-up to transmit the saved information to the monitoring center through the LoRaWAN gateway.

During the day, a charging circuit can be used from a photovoltaic panel mounted in the system, supplementing the energy requirements of the system for a longer lifetime. The whole system is designed for autonomous functionality, being able to be moved or reused for other monitoring areas without the need for interruptions from the power supply in case the power supply would be used directly from the switchgear. Using the GPS module, we can easily determine the position of the node and possibly assign new limits for the monitored parameters, changes that can be made from the control center.

The hardware platform from [27] with the new features added will need approximately 243 mA in active mode with SF7 and 352 mA with SF12. These consumption values are given for the worst-case scenario, when all the components from the platform are active at the same time.

For programming the platform, an open-source IDE programming environment is used. This is an easy way of integrating already existing functions and using existing libraries to perform communication within LoRaWAN protocol.

**Figure 5:**
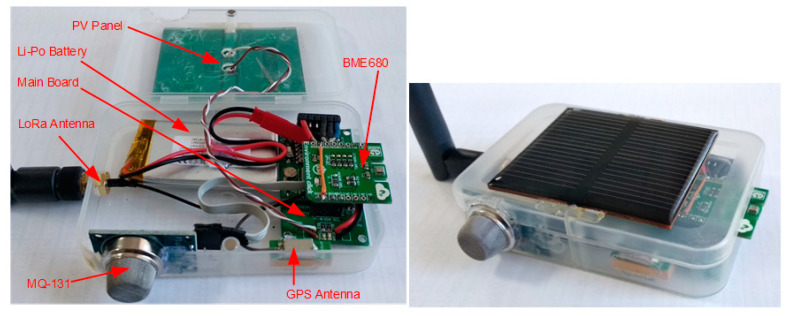
Hardware implementation of the MSMN node.

The hardware development of the node can be seen in Figure 5.

### 4.3. Communication Coverage of the Proposed Architecture

**Figure 6:**
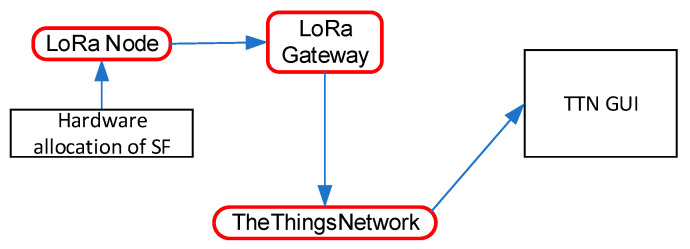
Communication architecture for the coverage estimation.

Given that this paper proposes a monitoring architecture for the smart grid concept, the proposed node must cover a large area for LoRaWAN communication. For proper communication, the gateways must cover the areas where switchgears for monitoring purpose are mounted. Thus, some tests are performed to ensure gateway communication and optimize the functionality parameters. The communication architecture is listed in Figure 6.

For this test, the node was programmed with a test code, with the communication spreading factor manually chosen with the use of some external hardware jumpers. Each time the packet was received, the message appeared in the TheThingsNetwork cloud IoT platform interface (TTN GUI). The used gateway was the one discussed in detail in [31], with an omnidirectional antenna from Taoglas with a 12 dBi gain and was placed in the Stefan cel Mare University Campus.

**Figure 7:**
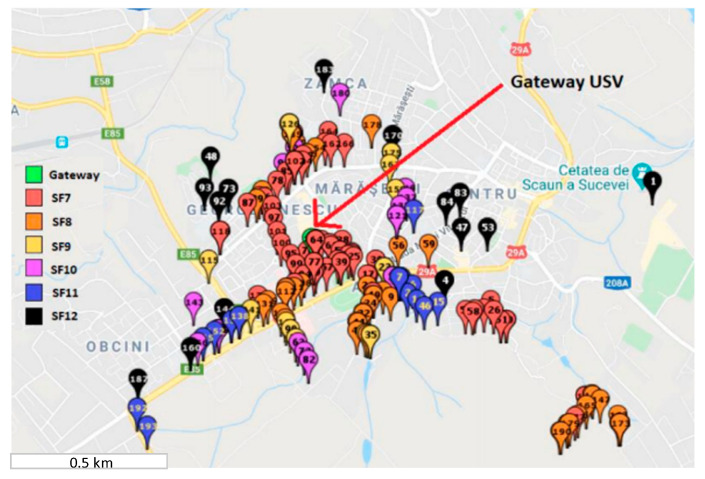
LoRaWAN coverage map measurement.

From the obtained results depicted in Figure 7, we can see that the communication range in the tested zone, which is an urban one, is strongly affected by the spreading factor, covering only approximately 2 km^2^ when all variations of the spreading factor are linked, from 7 to 12. Thus, to deploy a large-scale LoRaWAN network for the smart grid concept, we need to choose the gateways’ locations on the required infrastructure. The easiest method for this step is to use a wireless communication emulator. This network emulator must ensure the option for attenuation losses due to the geographical terrain variation, because LoRaWAN can be deployed for more than 10 km in the urban areas or more than 50 km in rural areas, where terrain variation with buildings and vegetation is available. Such a simulator is RadioPlanner [32] and it was used in this paper for the LoRaWAN coverage simulation. This software framework can deploy large-scale architectures using long-range communications like LoRa, GSM, LTE, UHF, or VHF.

**Figure 8:**
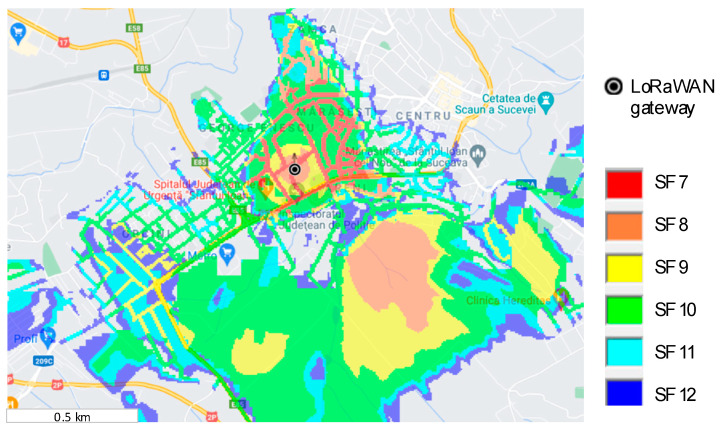
LoRaWAN coverage map simulation using RadioPlanner.

To ensure the simulation validation and calibration process, we chose the values measured with the test setup mentioned earlier. All the parameters were changed for the transmitter (the LoRaWAN node) and for the receiver (the LoRaWAN gateway), choosing the antenna pattern, gain, cable loss, beam tilt and the height where is mounted from the ground point. Also, for accurate results, some attenuation loss parameters were considered with the specifications from the ITU-R P.1812-4 report [33], which represents a path-specific propagation prediction method for point-to-area terrestrial services in the VHF and UHF bands. The simulated results are depicted in Figure 8.

## 5. Results and Discussion

To ensure the feasibility of the proposed sensor node, we chose some LV/MV switchgears from our regional electric distribution network, located in a neighborhood in the Suceava town. The monitored area is around 5 km^2^, it has 18 monitoring points, and it is an urban area where there are buildings and other steel/concrete structures, forest trees, and different elevations.

**Figure 9:**
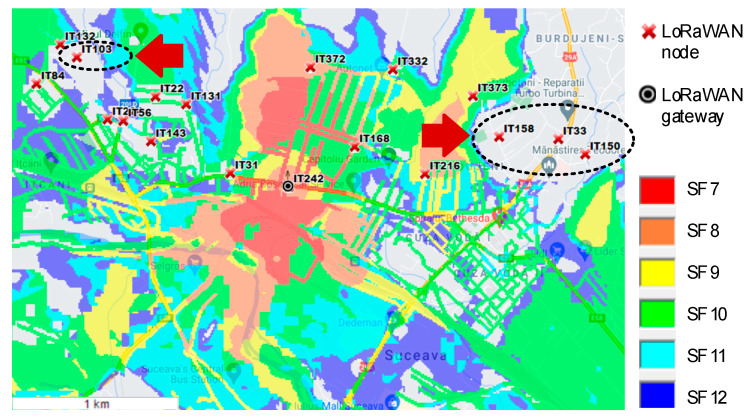
LoRaWAN Coverage Scenario 1.

The first step was to locate the best position of the LoRaWAN gateway. Taking the optimized parameters for the simulator, we made some gateway location estimates. The first scenario was placing the gateway in the middle of the monitored area (point IT242), with the results obtained in Figure 9.

**Figure 10:**
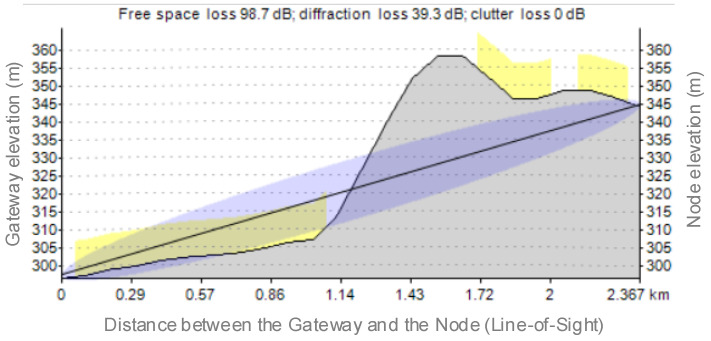
Losses due to the geographical terrain variation for Scenario 1.

From the first simulation, we can see that not all the LoRaWAN nodes were communicating with the gateway, even if the gateway was in the middle of the monitored area. This is due to the geographical terrain variation, which causes diffraction, free space, or clutter losses. A result from IT242 (the gateway location) and IT33 (one uncovered node location) is depicted in Figure 10.

**Figure 11:**
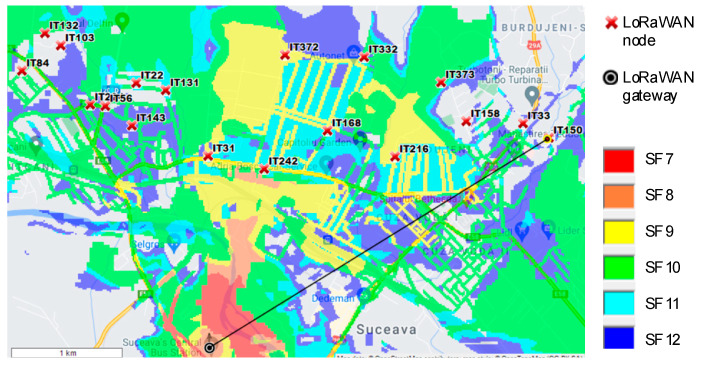
LoRaWAN Coverage Scenario 2.

The next scenario was made using other locations from the monitored area, but not linked directly to a LV/MV switchgear point. If we take the elevation parameter when making the simulation, we can find the best point for the gateway to be located (Figure 11).

**Figure 12:**
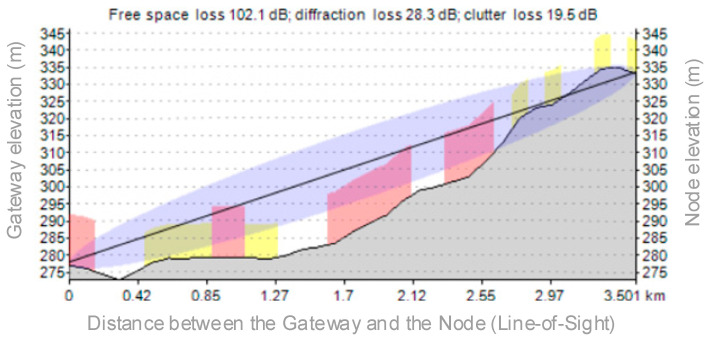
Losses due to the geographical terrain variation for Scenario 2.

Here we can see that all the monitored points are covered by the gateway, even if the distance between each monitored point and the gateway is greater than the previous scenario, covering a much larger area than the Scenario 1. Figure 12 reveals the elevation between the gateway location and the IT150 (the node location), with a LoS (line-of-sight) distance of 3.5 km. Even if the free space losses are greater than the previous scenario, the node can transmit data to the gateway using SF12 due to the elevation difference between the measuring points.

Once the gateway location is set, we can place the sensor nodes on the LV/MV switchgears for monitoring the environmental parameters.

**Figure 13:**
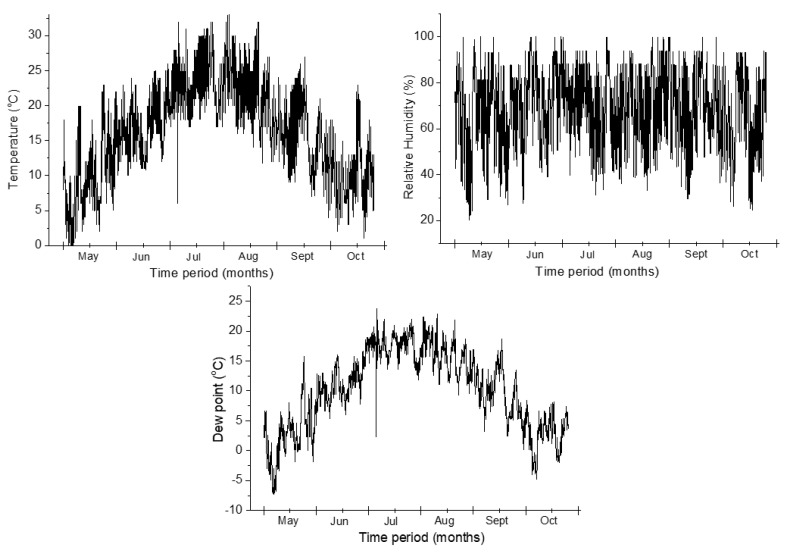
Environmental parameters from the MSMN (temperature, relative humidity, and dew point).

For testing purpose, we chose a faulty LV switchgear that needed maintenance on the cable side so that the transmitted values were accurate to the switchgear issues (appearance of the partial discharge inside), marked IT131 on the coverage map. The measurements were performed during a 6-month period, from May to October (Figure 13).

All the parameters mentioned in Section 4 were transmitted using the LoRaWAN communication protocol. The GPS coordinates were used to place the exact position of the nodes in the Google Maps view, so that each node can be easily identified. The temperature and the humidity were transmitted to obtain the dew point value inside the switchgear, to make an estimation regarding an eventual rising of its value that can cause partial discharge and degrade the conductor isolation. In technical terms, the dew point is the temperature at which the water vapor in a sample of air at constant barometric pressure condenses into liquid water at the same rate at which it evaporates. At temperatures below the dew point, the rate of condensation will be greater than that of evaporation, forming more liquid water. To obtain the exact value, some calculations are made using the equations from [34] as reference:

(1) $$t_{d}\approx t-\left(\frac{100-RH}{5}\right),$$

where *t_d_* is the dew point temperature measured in Celsius, *t* is the air temperature measured in Celsius, and *RH* is the relative air humidity in percentage. Listed below are the transmitted values and the dew point calculation according to Equation (1).

**Figure 14:**
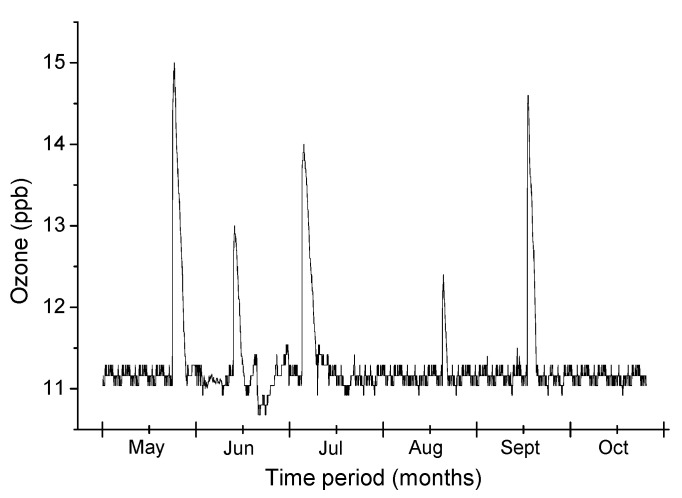
Ozone level received from the MSMN node.

Some issues regarding the switchgear can be noticed from the proposed MSMN node and this is the corona discharge effect, discussed in Section 4.2 of the paper. This is possible with the help of ozone values received from the node. In some periods, because of the increased relative humidity value, the dew point exceeds the air temperature, so water vapor condenses on the cable isolators. Thus, locally partial discharges occur for short periods, causing the increase of ozone levels. The obtained results received from the monitored switchgear can be seen in Figure 14.

The node was programmed to transmit the data three times a day (in the morning, in the afternoon, and in the evening); the rest of the time the node was in sleep mode, waking up only to sample the measurement parameters. This decision for transmitting the data was taken for power optimization, resulting an autonomous functionality node that does not need a continuous power supply, being a standalone LoRaWAN node. In the daytime, the solar panel will charge the node battery to increase power efficiency.

## 6. Conclusions

The maintenance of switchgear parameters from an electric grid is essential to avoid failures or blackouts. At present, the population is demanding of electricity, so it is necessary to find monitoring solutions for the distribution points.

The ever-increasing demand for electrical energy is also sustained by aggressive electrical vehicle technology integration to meet green energy certifications and carbon free emissions, according to EU regulations.

By using the internet of things infrastructure, we can remotely manage the electricity distribution points, receiving data that can predict any future failure points of the grid. By extending the concept, utility companies will be able to pick-up information from the network remotely and the network will even have the capability to self-heal in a cost-effective automatic manner.

In this paper, we present a smart grid monitoring architecture with a multi-sensor monitoring node from which we can collect data with the LoRaWAN communication protocol. The designed architecture was tested for a period of 6 months, revealing the feasibility and scalability of the system, and opening new directions in the remote prediction of failures and blackouts of the LV/MV switchgears on the electric grid. Furthermore, the monitoring solution is still transmitting data and we will continue to analyze its functionality in the cold wintertime. The measurements have been made during a period including the rainiest months in our region, May, and June. During these intervals, solar radiation was at an average compared to that of the sunniest days, which are in August. As per our laboratory tests with variable exposure to light, the sensor will operate with a minimum of 5 days per month of solar radiation.

This novel architecture can be integrated successfully into the smart city concept with smart grid applicability. Monitoring some normal environment parameters like air humidity and air temperature, we can obtain important information related to the condition of the network, allowing the evaluation of the performance level of the smart grid.

The novel designed multi-sensor monitoring node uses a modular platform with a high degree of reconfigurability that is also able to function in a standalone mode using state of the art communication protocols like LoRaWAN suitable for crowded city topologies.

## Figures and Tables

**Table 1:** Chirp length for each SF used in the LoRa modulation.

Spreading Factor	Chirp Length (bits)	Throughput (bps)
7	128	5469
8	256	3125
9	512	1758
10	1024	997
11	2048	537
12	4096	293

## Abbreviations

ICT	Information and communication technology
LPWAN	Low-power wide area network
LoRaWAN	Long-range wide area network
NB-IoT	Narrowband-internet of things
CPS	Cyber-physical system
IoT	Internet of things
PLC	Power line communication
TEV	Transient earth voltage
LV/MV	Low voltage/medium voltage
HFCT	High frequency current transformer
SCADA	Supervisory control and data acquisition
WSN	Wireless sensor network
CSS	Chirp spread spectrum
SNR	Signal to noise ratio
ISM	Industrial, scientific, medical
SRD	Short-range devices
SF	Spreading factor
MAC	Medium access control
ADR	Adaptive data rate
CR	Coding rate
ETSI	European telecommunications standards institute
FCC	Federal communications commission
DSSS	Direct sequence spread spectrum
LoS	Line-of-sight
MSMN	Multi-sensor measuring node
GSM/GPRS	Global system for mobile communications/general packet radio service
GPS	Global positioning system
MOS	Metal oxide semiconductor
MCU	Micro controller unit
LTE	Long-term evolution
UHF	Ultra-high frequency
VHF	Very-high frequency
MHz	Megahertz
kHz	Kilohertz
bps	Bits per second
mW	Milliwatt
mA	Milliampere
µA	Microampere
I2C	Inter-integrated circuit
mAh	Milliampere hour
t_d_	Dew point temperature
t	Air temperature
RH	Air humidity
